# Cancer stem cell metabolism

**DOI:** 10.1186/s13058-016-0712-6

**Published:** 2016-05-24

**Authors:** Maria Peiris-Pagès, Ubaldo E. Martinez-Outschoorn, Richard G. Pestell, Federica Sotgia, Michael P. Lisanti

**Affiliations:** The Breast Cancer Now Research Unit, Institute of Cancer Sciences, CRUK Manchester Institute, Paterson Building, University of Manchester, Manchester, UK; The Manchester Centre for Cellular Metabolism (MCCM), Institute of Cancer Sciences, CRUK Manchester Institute, Paterson Building, University of Manchester, Manchester, UK; The Sidney Kimmel Cancer Center, Philadelphia, PA USA

## Abstract

Cancer is now viewed as a stem cell disease. There is still no consensus on the metabolic characteristics of cancer stem cells, with several studies indicating that they are mainly glycolytic and others pointing instead to mitochondrial metabolism as their principal source of energy. Cancer stem cells also seem to adapt their metabolism to microenvironmental changes by conveniently shifting energy production from one pathway to another, or by acquiring intermediate metabolic phenotypes. Determining the role of cancer stem cell metabolism in carcinogenesis has become a major focus in cancer research, and substantial efforts are conducted towards discovering clinical targets.

Cancer, above all other diseases, has countless secondary causes.

But even for cancer, there is only one prime cause …: metabolism.

Otto Warburg

## The cancer stem cell model: *Omnis cellula e cellula*

Adult stem cells, in contrast to most cells in our body, which are differentiated and have a specific role, are rare cells that harbour unique biological properties such as a lack of differentiation and indefinite self-renewal. Stem cell asymmetrical division into one new stem cell and a committed progenitor, which can give rise to a functionally mature progeny, helps maintain tissue homeostasis [1].

Cancer is characterized by an unrestrained proliferation of malignant cells that are morphologically and functionally different. Two models have been proposed in order to explain this cellular diversity within tumours. The traditional, stochastic way of explaining cancer initiation and development is through sequential accumulation of mutations, each of which promotes the loss of specific tissue traits until dedifferentiation and regression into a more primitive phenotype occurs. According to this clonal evolution model, each cancer cell has a similar potential to grow a tumour. A second model, the cancer stem cell (CSC) hypothesis, postulates that a reduced group of stem-like cells is responsible for the development of the disease. Accordingly, tumours are hierarchically organized and sustained by a distinct self-renewal subpopulation of cancer cells. These tumour-initiating cells (TICs) with stemness properties are located at the apex of a pyramid and are responsible for the generation of a varied progeny of highly proliferative cells forming the bulk of the tumour [1, 2]. Both models are not mutually exclusive and can be viewed as integrated processes because CSCs can themselves undergo clonal evolution, through which a second more dominant population of CSCs may emerge. In addition, recent reports add more complexity to this scenario by demonstrating that cancer cells have a remarkable degree of plasticity. Indeed, it is thought that CSCs may arise from different cell types such as normal adult stem cells or differentiated cancer cells [2, 3].

**Fig. 1:**
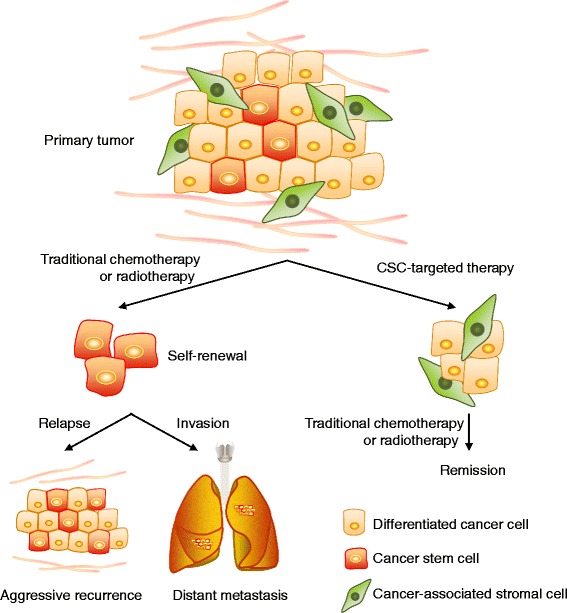
Potential impact of strategies that target cancer stem cells (CSCs) on the effectiveness of cancer treatment. Conventional cancer therapies result in a transient reduction of the tumour by killing non-stem cancer cells whilst failing to eliminate CSCs. Two major obstacles are limiting success in these cancer therapies: the ability of CSCs to survive cytotoxic treatments, and their potential to form metastases. The use of CSC specific inhibitors would reduce their therapy resistance and reduce relapse, and would prevent their spread, as the loss of stem cell properties reduces invasiveness and the capacity of disseminated cells to initiate distant secondary colonies

CSCs share numerous properties with normal stem cells besides their ability to renew themselves by remaining in an undifferentiated state: the expression of surface markers, such as CD44, CD133 or the enzyme aldehyde dehydrogenase (ALDH), the activation of particular cell signalling pathways, such as Wnt, Notch or Hedgehog, a relative quiescence or an active DNA repair capacity [2]. Given that CSCs are considered to be the source from which cancer cells arise, are therapy resistant and are responsible for metastatic dissemination, eliminating them could potentially achieve a permanent cure for the patient. In addition, if conventional therapy fails to kill CSCs, acting only against differentiated cancer cells, the tumour can eventually relapse [2]. The specific elimination of CSCs may thus represent one of the most important challenges of current cancer research (Fig. 1). Because of their similarity, an accurate distinction between CSCs and normal stem cells is needed, and once these differences are identified new therapies can be developed to eliminate CSCs without damaging normal cells. In particular, the metabolic features of CSCs might represent a promising target. In this review we summarize the latest findings and most significant discoveries on CSC metabolism.

## When metabolism takes over

Metabolic adaptation is believed to be one of the hallmarks of cancer cells [4]. The role of metabolism in cancer has become a dynamic field of research and a broad spectrum of novel strategies to target cancer metabolic pathways is emerging. However, the cellular heterogeneity present in tumours is not taken into account by most studies. It is important to highlight that different phenotypes such as hypoxic versus normoxic or quiescent versus proliferative will have substantially different metabolic requirements, which in turn may result in notably different responses to metabolic therapies. For example, CSCs – generally considered quiescent or slow-cycling compared with their differentiated cancer cell progeny – can re-enter into the cell cycle after exposure to radiotherapy, whereas most differentiated cells die or undergo cell cycle arrest [5].

Glycolysis is the enzymatic conversion of glucose into lactate, which concomitantly produces 2 molecules of ATP per molecule of glucose. In the presence of oxygen, cells generally adopt oxidative phosphorylation (OXPHOS) as the main pathway to produce energy, which is more efficient than glycolysis because it theoretically generates 36 molecules of ATP per molecule of glucose. Cancer cells can generate ATP via glycolysis even under normoxic concentrations in what is known as the Warburg effect. In fact, gycolysis can more rapidly produce ATP compared with OXPHOS in the presence of abundant levels of glucose [6]. Stem cells also rely more on glycolysis when compared with their differentiated offspring, which preferentially metabolizes glucose via mitochondrial respiration. Of note, the metabolic reprogramming of normal somatic cells into induced pluripotent stem (iPC) cells actually requires a shift from mitochondrial respiration to a metabolism that is mainly glycolytic [7], a switch which precedes the acquisition of pluripotency markers, suggesting that changes in metabolism occur before changes in stemness [8]. During differentiation, stem cells are also able to adjust their metabolic infrastructure, as they can rapidly shift from a preferentially glycolytic profile in undifferentiated cells to a more oxidative phenotype to generate the large amounts of energy needed for this process [9].

Studies of mitochondrial morphology and mitochondrial DNA levels indicate that stem cells have fewer mitochondria, which are less mature and relatively inactive compared with those of differentiated cells, resulting in reduced reactive oxygen species (ROS) levels [10]. Low amounts of ROS are actually needed to maintain quiescence and the self-renewal potential [11]. In sum, stem cells favour glycolysis and have less mitochondria, hence producing small amounts of ROS, which induce little mitochondrial DNA damage [9]. Thus, a glycolytic phenotype seems to be a shared feature of normal stem cells and differentiated cancer cells in culture. However, very few studies have directly investigated the metabolism of CSCs.

## The metabolic profile of cancer stem cells

### Are cancer stem cells mainly glycolytic?

Glucose seems to be an essential nutrient for CSCs, as its presence in the microenvironment significantly increases the amount of stem-like cells in the overall cancer cell population. On the other hand, glucose deprivation induces the depletion of CSCs in vitro [12]. However, the metabolic singularities of CSCs and the effects of different metabolites on CSC physiology remain largely unexplored, because the number of publications studying the metabolism of CSCs is small. Nevertheless, these studies indicate that CSCs have a distinctive metabolic phenotype compared with the bulk of the tumour and with normal stem cells, although there is so far no consensus on this [13].

Several reports suggest that CSCs are more glycolytic than other differentiated cancer cells in vitro and in vivo. These studies were performed in many tumour types including osteosarcoma, glioblastoma, breast cancer, lung cancer, ovarian cancer and colon cancer [14–18]. Glucose uptake, glycolytic enzyme expression, lactate production and ATP content are significantly increased in CSCs compared with their differentiated counterparts. This glycolytic phenotype seems to be linked to a decrease in mitochondrial oxidative metabolism [16–18]. Likewise, mutations in mitochondrial DNA and low mitochondrial DNA copy number have been associated with increased metastasis and poor prognosis [19, 20]. The mitochondrial DNA copy number not only affects the viability and functionality of the cell but also its differentiation potential. Cyclin D_1_ regulates stemness of cancer cells and mitochondrial DNA copy number [21, 22]. Also, during differentiation, the mitochondrial DNA copy number and the levels of mature cell gene expression patterns increase, whereas the expression of pluripotency genes such as *OCT4*, *TERT* and *MYC* decreases [23]. Instead, partial depletion of mitochondrial DNA increases the levels of these pluripotency genes.

### Or do they rely more on mitochondrial respiration?

**Fig. 2:**
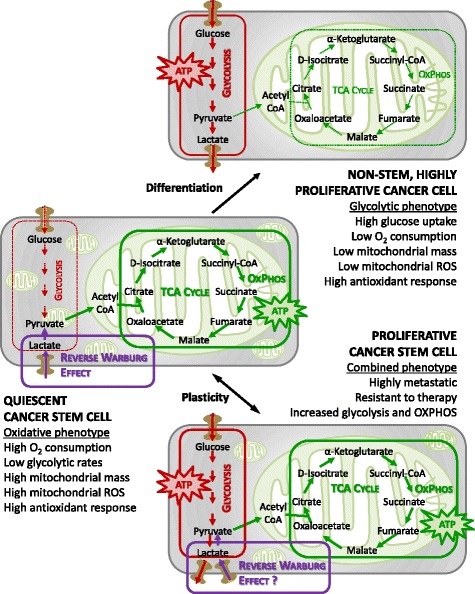
Bioenergetic pathways underlying CSC metabolism. In more differentiated cancer cells, the glycolytic phenotype might predominate over oxidative phosphorylation (*OXPHOS*). CSCs instead might rely more on an oxidative metabolism for their energy production. CSCs also appear to be metabolically plastic: when OXPHOS is blocked they can eventually develop resistance by acquiring an intermediate glycolytic/oxidative phenotype. *ROS* reactive oxygen species, *TCA* tricarboxylic acid

In clear contrast with these publications, growing evidence shows that CSCs have a preference for mitochondrial oxidative metabolism (Fig. 2). According to these other studies, CSCs are less glycolytic, consume less glucose, produce less lactate and maintain higher ATP levels than their differentiated progeny. Moreover, the mitochondria of CSCs have an increased mass and membrane potential, which is a reflection of mitochondrial function, higher mitochondrial ROS and enhanced oxygen consumption rates compared with the bulk of differentiated cancer cells, which generate their energy mainly via glycolysis [24–30]. Mitochondrial mass confers stem-like traits and is associated with metastatic potential and resistance to DNA damage [31]. Invasive migratory cancer cells also exhibit high mitochondrial metabolism via activation of a mitochondrial biogenesis mediator, the transcription co-activator peroxisome proliferator-activated receptor gamma co-activator 1 alpha (PGC1α) [32]. PGC1α has also been found overexpressed in circulating tumour cells [33], and its expression in a subset of human melanomas produces an increase in OXPHOS that is necessary for survival [34]. Moreover, PGC1α inhibition reduces the stemness properties of breast CSCs [24]. Oncogene ablation-resistant pancreatic cancer cells with features of CSCs also rely more on mitochondrial function to survive, and depend less on glucose and glutamine and more on pyruvate and palmitate to fuel the tricarboxylic acid (TCA) cycle [10]. Similarly, a population of CSCs isolated from ovarian cancer patients overexpressed genes associated with mitochondrial OXPHOS and fatty acid oxidation [28]. This oxidative phenotype seems to be related to the capacity to resist apoptosis in CSCs [35]. Despite mitochondrial ROS levels being high in these studies, total amounts of ROS are significantly lower in CSCs, which also show a more powerful antioxidant defence system compared with their progeny. A strong antioxidant response keeps ROS levels at bay, and helps in the maintenance of the stemness and tumourigenic capacities of CSCs, therefore contributing to therapy resistance [28, 36].

During differentiation under hypoxic conditions, CSCs from several tumour types are able to switch from an oxidative to a glycolytic metabolism in order to compensate for deficient mitochondrial machinery [37]. Likewise, CSCs might be able to regulate their differentiation via subtle changes of the redox status, with transitory bursts of ROS production that stimulate differentiation of CSCs towards their non-stem cancer cell counterparts [38]. Indeed, administration of antioxidants such as *N*-acetyl-cysteine (NAC) reduces ROS, suppressing the differentiation of CSCs and increasing metastatic burden [38, 39]. Furthermore, a recent study shows that epithelial stem-like cells apportion aged mitochondria asymmetrically between the two daughter cells with different fates. Those daughter cells that receive fewer old mitochondria maintain stem cell traits, whereas cells with a higher content of aged mitochondria are more prone to differentiate. This asymmetrical division of mitochondria requires the pertinent functioning of the mitochondrial fission machinery that spatially restricts old mitochondria to the perinuclear region of the mother cell [40]. Indeed, increased mitochondrial fission appears to be a characteristic of CSCs and its pharmacological or genetic inhibition leads to the loss of stemness traits and differentiation [40, 41]. Hence, the control mechanisms involved in the asymmetrical sorting of aged and young mitochondria, such as mitochondrial fission, also play an important role in CSC maintenance.

Finally, CSCs seem to be vulnerable to mitochondria-targeted drugs and the inhibition of OXPHOS seems to inhibit tumour formation [29, 42–44]. Treatment with metformin, an inhibitor of the OXPHOS complex I, induces the partial suppression of stemness traits, such as mammosphere formation, and in-vivo tumour growth delay, although the effects are not lasting and resistance to treatment is observed [29, 45, 46]. CSCs treated with a mitochondrial ROS inducer such as menadione do not become resistant, suggesting that increasing mitochondrial ROS levels to non-viable levels might be a better approach to eliminate CSCs [29]. Other studies indicate that the use of mitochondria-located antioxidants can prevent metastatic dissemination, suggesting that fine-tuning of oxidative stress to keep it below a critical threshold may be crucial for the maintenance of the CSC phenotype [47, 48]. According to symbiogenesis, which states that the origin of eukaryotic mitochondria was the engulfment of aerobic bacteria [49], the use of antibiotics has been postulated as an effective treatment to target mitochondrial mass and metabolism. Indeed, several approved antibiotics such as salinomycin, erythromycins, tetracyclines or glycylcyclines have already shown effects on CSC survival in preclinical models and in clinical studies via reduction of stemness properties [50–55]. Mitochondrial health is thus fundamental for the maintenance of CSCs and can be targeted for cancer therapy.

## The metabolic plasticity of cancer stem cells

One possible explanation for the discrepancies in CSC metabolism reported in the scientific literature may be found in the metabolic adaptability that CSCs show following microenvironmental fluctuations. For example, most in-vitro studies are carried out in non-physiological high glucose and oxygen concentrations, which favour a glycolytic phenotype. Ideally, the optimal experimental conditions to keep the metabolic traits of CSCs intact would be to isolate them directly from patients and analyse them immediately or within the first steps of in-vitro culture. In fact, when patient-derived, low-passage CSCs are used, OXPHOS seems to be the preferred metabolic pathway for the energy production of CSCs [56].

Breast CSCs in mouse and human tumours have a more glycolytic phenotype compared with their differentiated progeny [57]. CSCs from low-passage, patient-derived glioblastoma specimens relied more on OXPHOS than their differentiated progeny [56]. These glioma stem cells have high metabolic plasticity since they can switch to a glycolytic metabolism when OXPHOS is blocked [58]. Leukaemia stem cells also rely primarily on OXPHOS [26].

This observed adaptive metabolic plasticity might allow CSCs to survive in changeable, sometimes hostile, environments or unfavourable circumstances encountered during tumour progression, such as at metastatic sites. In support of this metabolic malleability scenario, some publications show that CSCs are able to switch to a glycolytic metabolism when OXPHOS is blocked [56, 59].

CSCs have been shown to adapt to starvation and hypoxia by upregulating glucose transporters and switching to a more glycolytic phenotype to outcompete their differentiated progeny [58, 60]. Hypoxia and changes in glucose concentration induce CSC enrichment, which is mediated by hypoxia-inducible factor 1 alpha (HIF1α) and the AKT/MTOR/beta-catenin (CTNNB1) stem cell regulatory pathway [61–63]. Inhibiting HIF1α in combination with anti-angiogenic therapy reduces CSCs in mouse models of breast cancer and holds promise to be an effective therapy in breast cancer, which is currently being investigated in clinical trials [64].

K-Ras ablation-resistant pancreatic cells with stem-like characteristics are unable to increase compensatory fluxes such as glycolysis following OXPHOS inhibition, despite being more resistant to nutrient deprivation and other environmental stresses. This lack of plasticity may be attributed to the shutdown of the K-Ras-driven metabolic programme [65]. Ont the contrary, in pancreatic CSCs the lack of plasticity seems to be independent of K-Ras. Instead, another oncogene, MYC, controls the observed limited metabolic adaptability of most metformin-treated CSCs, which undergo energetic crisis and die. However, MYC-dependent resistant clones eventually emerge [29]. These data suggest that oncogene activation is sufficient for the induction of a particular metabolic pathway in CSCs, and the extent of its effects on metabolic reprogramming may depend on the context; for example, on the differentiation status of the cell. Determining the mechanisms behind this specificity will be critical to understanding tumour heterogeneity and complexity.

Normal stem cells and iPS cells utilize glycolysis, while CSCs have the capacity to shift to OXPHOS and mitochondrial metabolism. One can speculate that differences in signalling pathways mediated by OCT3/4, SOX2, KLF4, MYC in iPS cells and by SHH, NOTCH, WNT, PTEN, MAPK, KRAS, HIF and TP53 in CSCs drives these divergent metabolic phenotypes. Understanding the metabolic differences between normal stem cells and CSCs and their regulators will be important for the process of developing novel therapeutics that metabolically target, but preserve, the key functions of normal stem cells.

### Glycolysis or mitochondrial respiration: is it really one or the other?

The CSC phenotype may not necessarily be uniform between cancer subtypes or even between tumours of the same subtype. For instance, the preferred metabolic pathway to produce energy may depend on the metastatic site, indicating extensive metabolic variability [66]. Moreover, different subpopulations of CSCs exhibit different metabolic patterns. Recent publications imply the existence of an epithelial-like (mesenchymal to epithelial transition (MET)) and a mesenchymal-like (epithelial to mesenchymal transition (EMT)) CSC phenotype, states that might be interconvertible [67, 68]. In breast cancer, MET CSCs are characterized by high ALDH activity and enhanced proliferative capacity, whereas EMT CSCs are identified by the expression of the CD44^hi^/CS24^–^ surface markers and a slow-cycling, quiescent state [68]. Mesenchymal-like EMT CSCs seem to favour glycolysis, and have a marked reduction in oxygen consumption, decreased mitochondrial mass and membrane potential, lower ROS production and higher antioxidant capacity compared with the epithelial-like fraction of CSCs [36, 67]. Indeed, CD44 acts as a metabolic modulator, by activating glycolysis under hypoxia. CD44 ablation reduces glycolysis and the antioxidant response, and moves the energy production to the mitochondria, with an increase in ROS [69]. Conversely, a proteomics study revealed that the ALDH-expressing population of CSCs express more glycolytic enzymes than the CD44^hi^/CS24^–^ CSCs [70]. Finally, a recent study shows that highly metastatic murine cancer cells enhance both glycolysis and OXPHOS pathways compared with cells with the same genetic background that lack metastatic potential [66]. Another report links metabolic plasticity to the acquisition of therapy resistance by showing that although most CSCs have limited metabolic malleability and predominantly rely on OXPHOS, a subpopulation of metformin-resistant CSCs is able to acquire a more adaptable intermediate glycolytic/respiratory phenotype. The metabolic phenotype of CSCs thus appears to be heterogeneous with distinct metabolic programmes activated in different subpopulations of cancer cells (Fig. 2).

These results suggest that the dual blockade of glycolysis and mitochondrial respiration may represent a better way to eradicate CSC heterogeneity than focusing exclusively on glycolysis inhibition or suppression of mitochondrial respiration. Indeed, combined inhibition of glycolysis and mitochondrial respiration has been shown to be effective in suppressing tumour growth and metastasis [71].

Genetic analysis of breast cancers has demonstrated different mutational profiles across the subtypes of breast cancer. For example, the most frequent genetic alteration found in luminal breast cancers is mutational activation of PI3K signalling [72]. In contrast, triple negative breast cancer (TNBC) almost always contain mutations in *TP53* and also frequently display deletions or epigenetic silencing of the *PTEN* tumour suppressor gene. In addition to *HER2* gene amplification, *HER2*-positive breast cancers frequently display deletions in *PTEN*, and indeed this is a likely cause of resistance to *HER2*-targeted therapies [73–75]. *BRCA1* germline mutations or epigenetic silencing of the *BRCA1* locus are most frequently associated with TNBCs [76]. All of these molecular alterations have been demonstrated to increase CSC frequency in pre-clinical models as well as in patient samples [3].

## The contribution of the microenvironment

The effects of the niche on CSC metabolism are also starting to be recognized. High catabolism in the microenvironment with NF-κB, HIF-1α and TGF-β activation coincides with glycolysis and ketogenesis, and promotes CSC features [77–80]. A model of reverse Warburg metabolism in which non-glycolytic stem-like cells may be fed by more differentiated glycolytic cells in normoxic conditions has also been observed in breast cancer [60]. Another study shows that EMT-induced cancer cells with CSC features have enhanced ability to utilize catabolites taken up from the extracellular microenvironment, such as the glycolytic end products pyruvate and lactate, the amino acids glutamine, glutamate and alanine, or ketone bodies, especially upon starvation, to support their mitochondrial energy production [81]. Indeed, glutamine, glutamate and alanine have been identified as EMT-associated metabolites in another report, which demonstrates that this oncometabolite signature correlates with poor survival in breast cancer [82]. Similarly, high lactate concentrations achieved by exogenous lactate administration increase the metastatic potential of breast cancer cells in vivo [83]. Finally, recent studies show that mitochondrial DNA transfer from host cells of the tumour microenvironment to tumour cells with compromised respiratory function re-establishes not only their mitochondrial respiration but also their tumour-initiating capacity and resistance to therapy [84, 85]. CSCs may thus enable the internalization of energy-rich nutrients or energy-producing mitochondrial components from the extracellular milieu to exploit in their own bioenergetic pathways. Although most studies focus on the interaction between cancer cells and host cells including immune cells, communication between heterogeneous populations of tumour cells might also be relevant.

Metabolic stresses also induce significant changes in non-malignant cells within tumours. T cells can preferentially develop into the immunosuppressive regulatory subtype (Tregs) following glucose restriction, which promotes tumour growth [86, 87]. Hypoxia alters interactions between breast CSCs and macrophages with transformation of macrophages to an immunosuppressive phenotype with upregulation of HIF-1α and HIF-2α [88, 89].

Conversely, inflammatory cytokines generated by the tumour microenvironment (such as IL-6 and IL-8) with activation of NF-κB induce glycolysis with activation of PI3K and AKT and stimulate CSC self-renewal, which then may promote tumour growth and metastasis [62, 90–92]. Drugs targeting IL-6 and IL-8 are being investigated as a therapeutic strategy specifically for CSCs [63]. Future studies will need to determine the effects of metabolic modulating therapies on the phenotype of different tumour cell populations and the role of metabolism modulation in the anticancer effects of drugs targeting CSC-sensitive cytokines and signalling pathways.

## Additional metabolic singularities of cancer stem cells

The role of cell metabolism has evolved into an active area of research during the last decade, with a strong focus on glucose metabolism. Unfortunately, scarce attention has been directed to amino acid and lipid metabolism. A recent report shows that pancreatic CSCs are glutamine dependent. Inhibiting glutamine availability by targeting glutaminase or glutamine oxaloacetic transaminase (GOT), accountable for the conversion of glutamine into oxaloacetate, reduces the expression of stemness genes, inhibits self-renewal and sensitizes CSCs to radiation therapy via accumulation of ROS in vitro and in vivo [93]. Colon adenocarcinoma circulating tumour cells are able to colonize hepatic tissue due to their elevated lysine catabolism, which reduces ROS levels, promotes self-renewal potential and activates the stemness-related Wnt signalling pathway [94]. Other studies show the importance of fatty acid metabolism and in particular the mevalonate pathway in the generation of CSCs [28, 65, 95]. Thus, very little is known regarding the role of protein and fatty acid metabolism in CSC biology, and further investigations will be required to elucidate their contribution to such CSC traits and their relationship with glucose metabolism. Likewise, a strong dependence of CSCs on other catabolic processes such as autophagy, which makes them more resistant to nutrient deprivation and other stresses [96], should be further investigated. An entire world of metabolic pathways might be awaiting future discovery.

## Final remarks and challenges ahead

Over the years, substantial evidence for the existence of CSCs has strengthened the view that these cells are accountable for cancer development. CSCs renew themselves and at the same time generate progenitors that lose their stemness, ultimately giving rise to the bulk of the tumour. In addition, the last decade of research highlights that metabolism is not a mere player subordinated to CSC physiology, but actually may orchestrate it. Given that changes in metabolism precede changes in stemness, a perturbation in the metabolic phenotype of CSCs could be essential for the acquisition of the CSC state. Conventional therapy targets rapidly proliferating cancer cells that make up the bulk of the tumour without necessarily having an effect on the CSC population. CSCs possess special metabolic traits that distinguish them from the bulk of the tumour and that may constitute the basis for the development of new therapeutic strategies to eradicate them. From a clinical point of view, targeting the particularities of CSC metabolism is more likely to translate into permanently curing cancer or at least providing long-term disease-free survival, because these cells are responsible for resistance to therapy and metastasis, the main cause of cancer-related deaths. The interest in exploiting CSC metabolism for drug targeting is therefore gaining ground. However, a well-defined portrait of the singularities of CSC metabolism still needs to be depicted and CSC metabolism remains a controversial issue, with studies supporting a glycolytic phenotype of CSC and others stating that CSC metabolism is mainly oxidative.

Many other unresolved issues need to be addressed. Elucidating the differences in metabolism between CSCs and non-stem cancer cells, and between CSCs and normal progenitor stem cells, will be crucial to develop new therapies and may reveal new ways to distinctively target these TICs. Whereas normal stem cells rely more on glycolysis, CSCs might depend more on mitochondrial oxidative metabolism. If this is the case, why would the stemness state of cancer cells require a different metabolic state than normal stem cells? In contrast with normal physiological development, tumourigenesis tends to be highly disorganized and cancer metabolic malleability could provide a niche more prone to CSC survival. Nevertheless, the stability or plasticity of the CSCs phenotype needs to be verified. Are CSCs really able to metabolically adapt depending on microenvironmental fluctuations? Is the CSC population metabolically heterogeneous or does it exhibit different degrees of stemness-related phenotypes? During tumourigenesis, characteristics of the CSCs might mutate, and distinct CSC populations could eventually emerge in what it would be a metabolically changeable or versatile target. In such a scenario, future therapies designed to eradicate CSCs via targeting their metabolism might need to simultaneously block glycolysis and mitochondrial respiration.

We therefore need to overcome multiple obstacles before we can effectively eliminate CSCs. First of all, to properly recognize CSCs and differentiate them from other cell types, greater efforts should be made towards the identification of specific CSC markers, because none of the markers so far defined is unique for CSCs. Likewise, a combination of markers could greatly improve the purity of CSCs for research purposes. Finally, it is important to note that CSCs are settled in a niche formed by multiple other cell types and cancer heterogeneity is clearly more complex than originally thought. Hence, studying the metabolism of CSCs in experimental settings that do not reflect the heterogenic architecture of tumours such as the absence of a pertinent microenvironment is not ideal. Better models that preserve the CSC physiological state and structure should be developed.

Despite the limited information we currently have on the role of metabolism in the ability of CSCs to self-renew, initiate tumours, metastasize and survive therapy, targeting CSCs by blocking their metabolic singularities holds great potential in improving current cancer treatments. In practice, combinational treatments involving both a standard cytotoxic therapy and a CSC-targeted therapy will probably be required to ablate all cancer cells (Fig. 1).

## Abbreviations

ALDH: aldehyde dehydrogenase
CSC: cancer stem cell
EMT: epithelial to mesenchymal transition
HIF: hypoxia-inducible factor
iPS: induced pluripotent stem
MET: mesenchymal to epithelial transition
NAC: *N*-acetyl-cysteine
OXPHOS: oxidative phosphorylation
PGC1α: peroxisome proliferator-activated receptor gamma co-activator 1 alpha
ROS: reactive oxygen species
TCA: tricarboxylic acid
TIC: tumour-initiating cell
